# Capacitive versus Overlap Decoupling of Adjacent Radio Frequency Phased Array Coil Elements: An Imaging Robustness Comparison When Sample Load Varies for 3 Tesla MRI

**DOI:** 10.1155/2020/8828047

**Published:** 2020-12-15

**Authors:** Michael J. Beck, Dennis L. Parker, J. Rock Hadley

**Affiliations:** Department of Radiology and Imaging Sciences, Utah Center for Advanced Imaging Research, University of Utah, Salt Lake City 84132, USA

## Abstract

Phased array (PA) receive coils are built such that coil elements approximate independent antenna behavior. One method of achieving this goal is to use an available decoupling method to decouple adjacent coil elements. The purpose of this work was to compare the relative performance of two decoupling methods as a function of variation in sample load. Two PA receive coils with 5 channels (5-ch) each, equal outer dimensions, and formed on 12 cm diameter cylindrical phantoms of conductivities 0.3, 0.6, and 0.9 S/m were evaluated for relative signal-to-noise ratio (SNR) and parallel imaging performance. They were only tuned and matched to the 0.6 S/m phantom. Simulated and measured axial, sagittal, and coronal 5-ch PA coil SNR ratios were compared by dividing the overlap by the capacitive decoupled coil SNR results. Issues related to the selection of capacitor values for the two decoupling methods were evaluated by taking the ratio of the match and tune capacitors for large and small 2 channel (2-ch) PA coils. The SNR ratios showed that the SNR of the two decoupling methods were very similar. The inverse geometry-factor maps showed similar but better overall parallel imaging performance for the capacitive decoupled method. The quotients for the 2-ch PA coils’ maximum and minimum capacitor value ratios are 3.28 and 1.38 for the large and 3.28 and 2.22 for the small PA. The results of this paper demonstrate that as the sample load varies, the capacitive and overlap decoupling methods are very similar in relative SNR and this similarity continues for parallel imaging performance. Although, for the 5-ch coils studied, the capacitive decoupling method has a slight SNR and parallel imaging advantage and it was noted that the capacitive decoupled coil is more likely to encounter unbuildable PA coil configurations.

## 1. Introduction

Simultaneous and independent imaging with multiple surface coils, also known as phased array (PA) coils, brings with it many benefits [1]. The benefits, cited by Roemer et al., are increased surface coil array field of view (FOV) while maintaining the local high signal-to-noise ratio (SNR) of each individual coil element [2]. Roemer proposed the first method of combining individual images on a pixel by pixel basis to form a composite image using optimal weights and phases; these were derived assuming independent antenna behavior (zero mutual coupling between coil elements).

PA coils can still be used even when strong coupling exists between coil elements if the magnitude and phase of all the mutual coupling between coil elements are known. The coupling information can be used to remove any signal and noise that was transferred from one coil element to another through the mutual coupling [3]. The resulting signals will appear as if they were received from coil elements with independent antenna behavior. However, it is much easier to design and build a PA coil that has had the coupling minimized between coil elements. When a coil element is decoupled from other coil elements, its tune can be altered without affecting the tune of the remaining coil elements. This is not the case with coupled PAs where the other coil elements’ tunes can be largely affected, making PAs with large numbers of coil elements impractical to tune.

There are many methods currently used for decoupling [3]. One of the most common techniques to minimize mutual inductance between adjacent coil elements is implemented by partially overlapping them or the overlap decoupled (OD) method [2]. An alternative magnetic decoupling technique is implemented by joining surface coils together with a common rung and using a capacitor, on that common rung, to eliminate the mutual inductance or the capacitive decoupled (CD) method [4–7]. These types of PAs are commonly referred to as ladder coils [6, 8–10]. Additional techniques for receive PA coils include canceling flux with inductors [11, 12], the use of decoupling shields [13, 14], passive resonators [15], and self-decoupled coils [16]. A review of the different decoupling methods and their advantages and disadvantages are given by Hui et al. [3].

The stated decoupling methods are for adjacent coil elements. For nonadjacent coil elements, the effects of mutual coupling can be reduced by using mismatched preamplifiers. These preamplifiers reduce magnetic flux linkage by helping to create a high impedance at the input of the coil which suppresses the magnetic flux generating current [17]. This method is typically used in addition to the decoupling methods already mentioned.

Although there have been papers that present the different decoupling methods, a direct comparison of the different methods has not been published. It remains to be shown whether one method has an advantage in SNR, is robust to variations in load, or has issues related to construction.

This work compared the simulated and measured SNR and parallel imaging performance of a 5-channel (5-ch) CD PA coil and 5-ch OD PA coil as a function of coil loading. The 5-ch PA coils were formed around cylindrical phantoms, and the robustness in imaging performance between these two common decoupling methods was investigated. Lastly, the buildability of PA coils utilizing either of these decoupling methods was explored using small and large 2-channel (2-ch) PA coils.

## 2. Theory

As stated previously, Roemer et al. derived the optimal weights and phases for the NMR PA assuming no magnetic coupling [2]. Magnetic coupling between coil elements not only results in frequency splitting, but sharing of both signal and noise (crosstalk) [2, 18]. If crosstalk is not compensated for before combining individual images into a single composite image, when using Roemer’s method of combining, image quality may be adversely affected [18, 19]. For this reason, it is imperative that magnetic coupling be minimized as much as possible.

### 2.1. Overlap Decoupled Theory.

Decoupling by overlapping adjacent coil elements can be understood by Faraday’s law of induction and Lenz’s law:

(1) $$\varepsilon_{21}=-\frac{\partial \Phi_{21}}{\partial t}=-\int_{\text{Surface }}\frac{\partial B{\left(r,t\right)}_{1}}{\partial t}\cdot \text{d}A_{2},$$

where *ε*, Φ, *B*, and *A* are the electromotive force, magnetic flux, magnetic field, and surface area, respectively. Crosstalk between coils 1 and 2 is dictated by Faraday’s and Lenz’s laws. From Faraday’s law, an equation for mutual inductance follows (equation (2)) [20] with Φ, *M*, and *I*:

(2) $$M_{21}=\frac{\Phi_{21}}{I_{1}},$$

being the magnetic flux, mutual inductance, and electric current, respectively. The magnitude of the mutual inductance is a function of the geometry of the coil elements.

The mutual impedance through which signal and noise is transferred between coils is shown in [4, 21]:

(3) $$Z_{21}=R_{21}+j\omega M_{21}.$$

There is a resistive component, *R*_21_, associated with correlated noise and a reactive component, *M*_21_, through which crosstalk occurs [4, 18, 21]. The resistive component will be discussed below, but for equation (2), the mutual inductance, *M*_21_, will equal zero when the net magnetic flux is equal to zero with nonzero current flowing in loop 1. When observing equation (1), this is possible because coil element 1 can generate magnetic field components, perpendicular to the surface area of coil element 2, which are both positive and negative when the two coils are overlapped. As a result, the sum of the integral in equation (1) can equal zero and two coil elements can be decoupled by elimination of the mutual inductance. Roemer et al. found the decoupling distance between identical circular coil elements is ~75% of the diameter when measured from the center of each coil element [2]. In practice, there is also a mutual capacitance, but the reactance of this mutual capacitance is typically small compared to the reactance of the mutual inductance and it was assumed to be equal to zero [16].

### 2.2. Capacitive Decoupled Theory.

Capacitive decoupling utilizes a capacitor to cancel out the reactance of the mutual inductance between adjacent coil elements as shown in equation (4) [4]. Adjacent CD coil elements are not overlapped but instead share a common rung. Crosstalk between adjacent coil elements is eliminated by adding a capacitor on the shared rung which forms a reactance that can cancel the reactance of the mutual inductance. The greatest amount of coil-to-coil decoupling results when the magnitude of the capacitor reactance equals the magnitude of the mutual inductance reactance at the resonant frequency:

(4) $$Z_{21}=R_{21}+j\omega M_{21}+\frac{1}{j\omega C_{21}}.$$

### 2.3. Electric Field and Correlated Noise.

Using the principle of reciprocity, electric fields generated by each PA coil element interact with the sample resulting in correlated sample noise. This correlation is characterized by the resistance, *R*_21_, of the mutual impedance as stated previously. These resistances represent the covariance of the noise between coil elements. An in-depth study of the effect of correlated noise is presented by Hayes et al. [22]. These resistances do not result in frequency shifting and signal/noise sharing (crosstalk) [2] but can limit SNR [22].

## 3. Methods

A simulated analysis was performed, using SNR and parallel imaging data, to determine which decoupling method would be more robust to changes in sample load using 5-ch PA coils. The 5-ch PA coils were constructed and SNR and parallel imaging data were measured to compare against the simulation data. In addition, using large and small 2-ch PA coils, the amount of coil element capacitor value variation for each decoupling method was compared to determine which method would most likely encounter unbuildable PA coil configurations due to unrealizable capacitance values. All simulations were performed using Computer Simulation Technology (CST), Microwave Studio (MWS), and CST Design Studio (DS) cosimulation.

### 3.1. Simulations of 5-ch Coils.

SNR and parallel imaging performances were calculated using two 5-ch PA coils decoupled by one of each described decoupling methods. They were tuned and matched to a 0.6 S/m phantom and then, without making any changes to tune and match, numerically measured on phantoms with conductivities of 0.3, 0.6, and 0.9 S/m.

SNR was determined using signal and noise voltage calculations. The signal voltage was calculated using CST-MWS from the magnetic field of each coil element, as generated by one ampere of current in the coil element [23]. B1^−^ field values were calculated using equation (5b) [24]:

(5a) $$B_{1}^{+}=\frac{B_{1x}+jB_{1y}}{2},$$

(5b) $$B_{1}^{-}=\frac{{\left(B_{1x}-jB_{1y}\right)}^{*}}{2}.$$

The transmit coil magnetic field, B1^+^ from equation (5a), would normally be used in flip angle determination; however, the transmit coil was not simulated in CST-MWS and a constant 90° flip angle was assumed. The magnetization was calculated using equation (6a) with *M*_*o*_, *ρ, γ*, ħ*, B*_*o*,_ *K*_*B*_ *T, M*^+^, *α, Tr, T*1, and *T*2 being the magnetization at equilibrium, proton density, gyromagnetic ratio, Planck’s constant divided by 2*π*, static magnetic field strength, Boltzmann’s constant, temperature (Kelvin), magnetization (positive rotating frame), flip angle, repetition time, longitudinal relaxation time, and transverse relaxation time, respectively [25]. Lastly, equation (7) was used to calculate the signal [24]:

(6a) $$M_{o}=\frac{\rho \gamma^{2}\hbar^{2}B_{o}}{4K_{B}T},$$

(6b) $$M^{+}=M_{o}\frac{\left(1-e^{-Tr/T1}\right)}{1-\text{cos}\;\alpha e^{-Tr/T1}}\text{sin}\;\alpha e^{-t/T2^{*}},$$

(7) $$V_{s,i}=\omega M^{+}{\left(B_{1}^{-}\right)}^{*}.$$

The noise voltage was calculated using equation (8) where *ψ* Δf and *N* are the noise covariance matrix and bandwidth (inverse of dwell time), and *N* is the product of the number of phase and frequency encodes. The noise covariance matrix was extracted from CST-DS by removing the match capacitors and then measuring the real part of the z-parameters [26]. The noise covariance matrix was scaled to obtain SNR values more consistent to what would be measured on the MRI scanner using the method outlined by Kellman et al. [27]. Equations (7) and (8) are both in the image domain and equation (8) would give the noise in k-space if not for dividing by *N*. Equation (9) was used to combine images from individual channels into a single SNR composite image [2]:

(8) $$V_{n,i,j}=\frac{4K_{B}T\psi_{i,j}\Delta f}{N},$$

(9) $${\text{SNR}}_{\text{composite }}=\sqrt{V_{s}^{T}V_{n}^{-1}V_{s}^{*}}.$$

**Figure 1:**
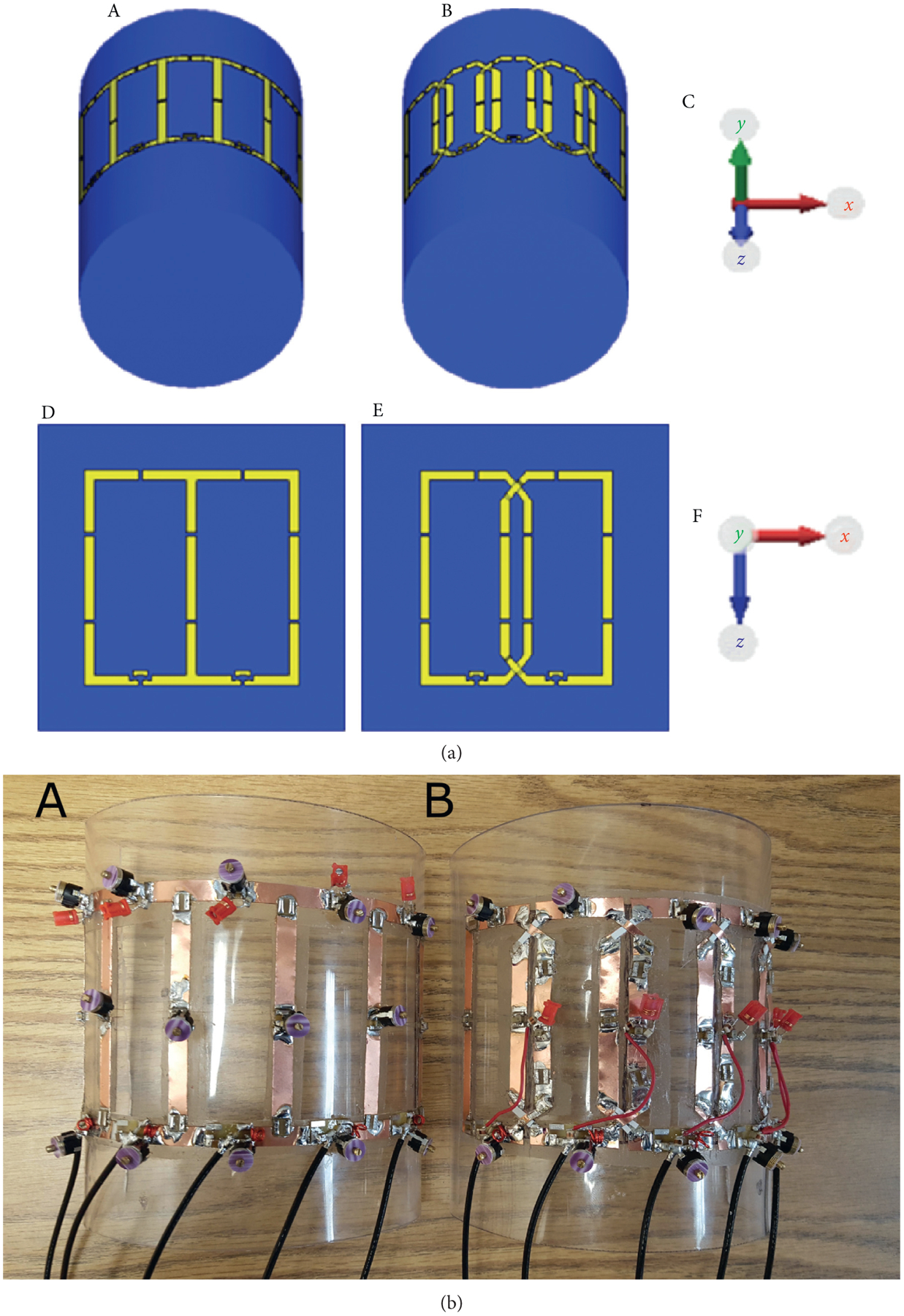
(a) Coil and phantom setup examples, coils and phantoms are not to scale. (A) 5-ch CD PA coil used for SNR and 1/g-factor map simulations. (B) 5-ch OD PA coil. (C) Axis showing orientation of (A) and (B). (D) Large 2-ch CD PA coil used for the coil element capacitor value variation. (E) Large 2-ch OD PA coil. (F) Axis showing orientation of (D) and (E). (b) 5-ch PA coils used to acquire measurement data. (A) 5-ch CD PA coil. (B) 5-ch OD PA coil.

Parallel imaging comparisons were made between the 5-ch CD and OD PA coils by calculating inverse geometry-factor maps (1/g-factor) with acceleration rates of ×2–×5 [28]. Each 5-ch PA coil was tuned and matched to the 0.6 S/m phantom and, without changing any passive component values, 1/g-factor map calculations on the 0.3, 0.6, and 0.9 S/m phantoms were made. The 5-ch PA coil and phantom setups for the parallel imaging comparisons were the same as those for the SNR comparisons (see Figure 1).

The two 5-ch PA coils were modeled with the following shared physical constraints, using center-to-center conductor measurements: the same overall *x* and *z* outer dimensions of 15 cm × 8 cm, respectively, copper trace width of 5 mm, and five channels per PA coil. The 5-ch CD PA coil had coil element *x* and *z* dimensions of 3 cm × 8 cm, respectively, for all coil elements, while the 5-ch OD PA coil elements all had *x* and *z* dimensions of 3.6 cm × 8 cm, respectively. The PA coils were also constrained to have the same outer dimensions. Each coil element had 4 equally spaced capacitors around the perimeter, this includes the match capacitor. See Figure 1 for coil configuration example and axis directions.

Three cylindrical phantoms were simulated with each 5-ch PA coil. The phantom properties used were water as the material, 78 as the dielectric constant, and 0.3, 0.6, or 0.9 S/m as the water conductivities. The conductivities chosen represent the typical conductivity ranges seen in the human body [29]. Each phantom had a diameter and length of 12 and 20 cm, respectively. A 3 mm separation was maintained between each PA coil and phantom without a former.

In the setups used for the 5-ch PA coil simulations there were approximately 24 million hexahedral mesh cells for the 5-ch CD PA coil simulations and approximately 14 million hexahedral mesh cells for the 5-ch OD PA coil simulations. All simulations had the following settings: an accuracy of −80 dB, online AR-filter analysis with a max steady state error of 0.001, minimum mesh cell size of 0.1 mm, max mesh cell size of 8.4 mm near the model, max cell size of 7.5 cm far from the model, and no adaptive mesh refinement. The equilibrate ratio was 1.1 and 1.25 for the 5-ch CD and 5-ch OD PA coils, respectively. Preamplifier decoupling was implemented by including the inductor across the match capacitor and setting the port impedance to 3 ohms. All coil elements of the 5-ch PA coils were only ever tuned and matched to 50 ohms on the 0.6 S/m phantom.

### 3.2. Imaging Measurements of 5-ch Coils.

A 5-ch CD PA coil, a 5-ch OD PA coil, and three phantoms were constructed to gather measurement data. The dimensions and layout of the constructed 5-ch PA coils were equivalent to the simulated 5-ch PA coils, except for the physical coils were constructed on a 3 mm thick cylindrical polycarbonate former to provide the 3 mm coil to phantom separation. The desired active decoupling magnitude was achieved by placing each active decoupling inductor across a tune capacitor. Solely placing the active decoupling inductors across match capacitors resulted in insufficient active decoupling magnitudes. All coil elements were only ever tuned and matched to 50 ohms on the 0.6 S/m phantom and attached to a preamp via a 25 cm cable and a pi phase shifter that completed a 180° phase shift for optimal preamp detuning. Three phantoms each with a 12 cm diameter and a length of 20 cm were filled with phantom solutions containing 1.955 g CuSO_4_ × H_2_0 combined with 1.094, 3.020, and 4.915 g NaCl for conductivities of 0.3, 0.6, and 0.9 S/m, respectively, at 20° Celsius.

SNR and parallel imaging measurements were acquired using a MAGNETOM 3T Trio (Siemens Healthcare, Erlangen, DE) MRI scanner. The measurements were made with 2D gradient echo (GRE) axial, sagittal, and coronal images with a TE/TR = 4.0/500 ms, flip angle = 90°, matrix size = 320 × 320, FOV = 300 × 300, slice thickness = 5 mm, bandwidth = 256 hertz/pixel, and noise-only images using the same sequence with the RF transmit voltage set to 0 volts and TR = 50 ms. The axial, sagittal, and coronal images were acquired by positioning each slice so it will image through the center of the phantom.

### 3.3. Coil Element Capacitor Value Variation of 2-ch Coils.

Simulations were performed to compare how much variation in match, decoupling (CD configuration only), and tune capacitor values there was for each tuned and matched coil element of the 2-ch CD and OD PA coils using small and large coil element configurations. The 2-ch CD PA coils had two predetermined capacitance value types, determined by the load (match capacitor) and mutual inductance (decoupling capacitor), and the 2-ch OD PA coils only had one capacitor value type predetermined by the load (match capacitor).

**Figure 2:**
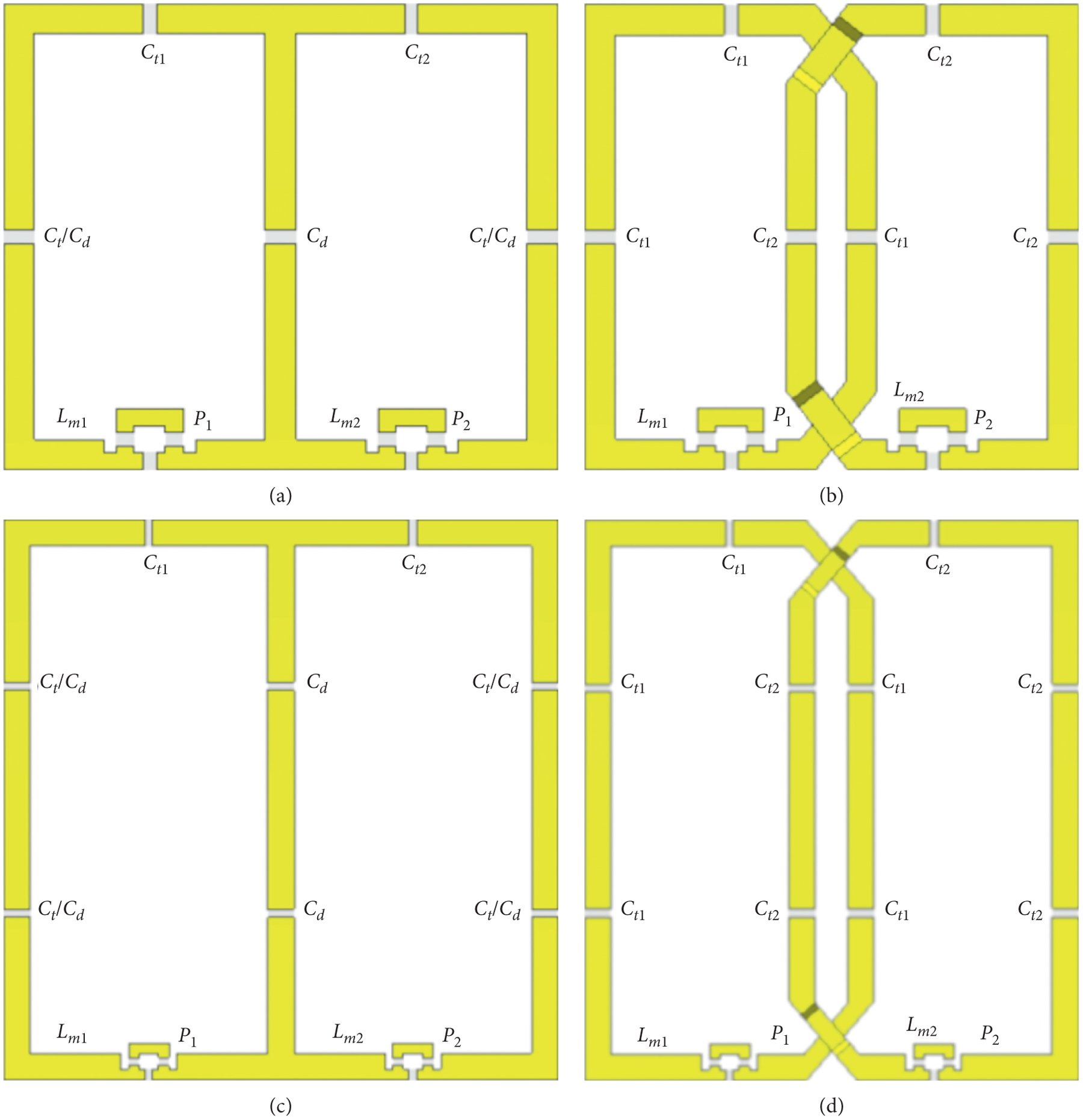
Schematic showing component placement on the small and large 2-ch PA coils. (a) Small 2-ch CD PA coil. (b) Small 2-ch OD PA coil. (c) Large 2-ch CD PA coil. (d) Large 2-ch OD PA coil. The inductors (*L*_m1_ and *L*_m2_) and excitation ports (P_1_ and P_2_) are labeled as well. The relative size of the small and large 2-ch PA coils is not to scale.

The coil element capacitor value variation comparison was made using four 2-ch PA coils: a small and large 2-ch CD PA coil, and a small and large OD PA coil. The coil element size of each small 2-ch PA coil (small PA pair) was constrained to be equal independent of the decoupling method. The same is true for the coil element size of each large 2-ch PA coil (large PA pair). This constraint was met by allowing the outer dimensions of each PA pair to be different. The coil element dimensions for the small PA pair were 3 × 5 cm in the *x* and *z* directions, respectively, with trace widths of 3.5 mm. The coil element dimensions for the large PA pair were 5 ×10 cm in the *x* and *z* directions, respectively, with trace widths of 5 mm. Coil element dimensions were measured from the center of each trace. Each coil element of the small PA pair had 4 equally spaced capacitors around the perimeter while there were 6 for the large PA pair, which includes the match capacitor. See Figure 2 for coil configurations and Figure 1 for axis directions.

The small and large 2-ch PA coils were simulated in two configurations (tune *C*_d_ and *C*_t_). Tune *C*_d_ configuration is defined by constraining all rung capacitance (*C*_d_ and *C*_t_/*C*_d_) values to be equal while tune *C*_t_ configuration is defined by constraining the outer rung capacitance (*C*_t_/*C*_d_) values and tune capacitance (*C*_t1_ and *C*_t2_) values to be equal. The *C*_d_ tune condition is an attempt to demonstrate the limitations that would occur in the inner elements of a larger coil array. In this simple 2-ch geometry, we simulate a larger number of coil elements by requiring the capacitance *C*_t_/*C*_d_ to be equal to the decoupling capacitance, *C*_d_. The capacitor labels can be referenced in Figure 2.

A single simulated rectangular phantom was used for the small and large 2-ch PA coils. The phantom properties used were water as the material, 78 as the dielectric constant, and 0.6 S/m as the conductivity. The rectangular phantom was simulated with *x*, *z*, and *y* dimensions of 15 × 15 × 10 cm, respectively.

In the setup used for the 5-ch simulations, there were approximately 8 to 9 million hexahedral mesh cells for the small and large 2-ch CD PAs, respectively, and approximately 10 to 11.5 million hexahedral mesh cells for the small and large 2-ch OD PAs, respectively. All simulations had the following settings: an accuracy of −80 dB, online AR-filter analysis with a max steady state error of 0.001, minimum mesh cell size of 0.1 mm, max mesh cell size of 8.4 mm near the model, max cell size of 7.5 cm far from the model, no adaptive mesh refinement, and an equilibrate ratio of 1.1. Preamplifier decoupling was implemented by including the inductor across the match capacitor and setting the port impedance to 3 ohms. All coil elements of the 2-ch PA coils were tuned and matched to 50 ohms.

## 4. Results

### 4.1. 5-ch PA Coils: SNR.

**Figure 3:**
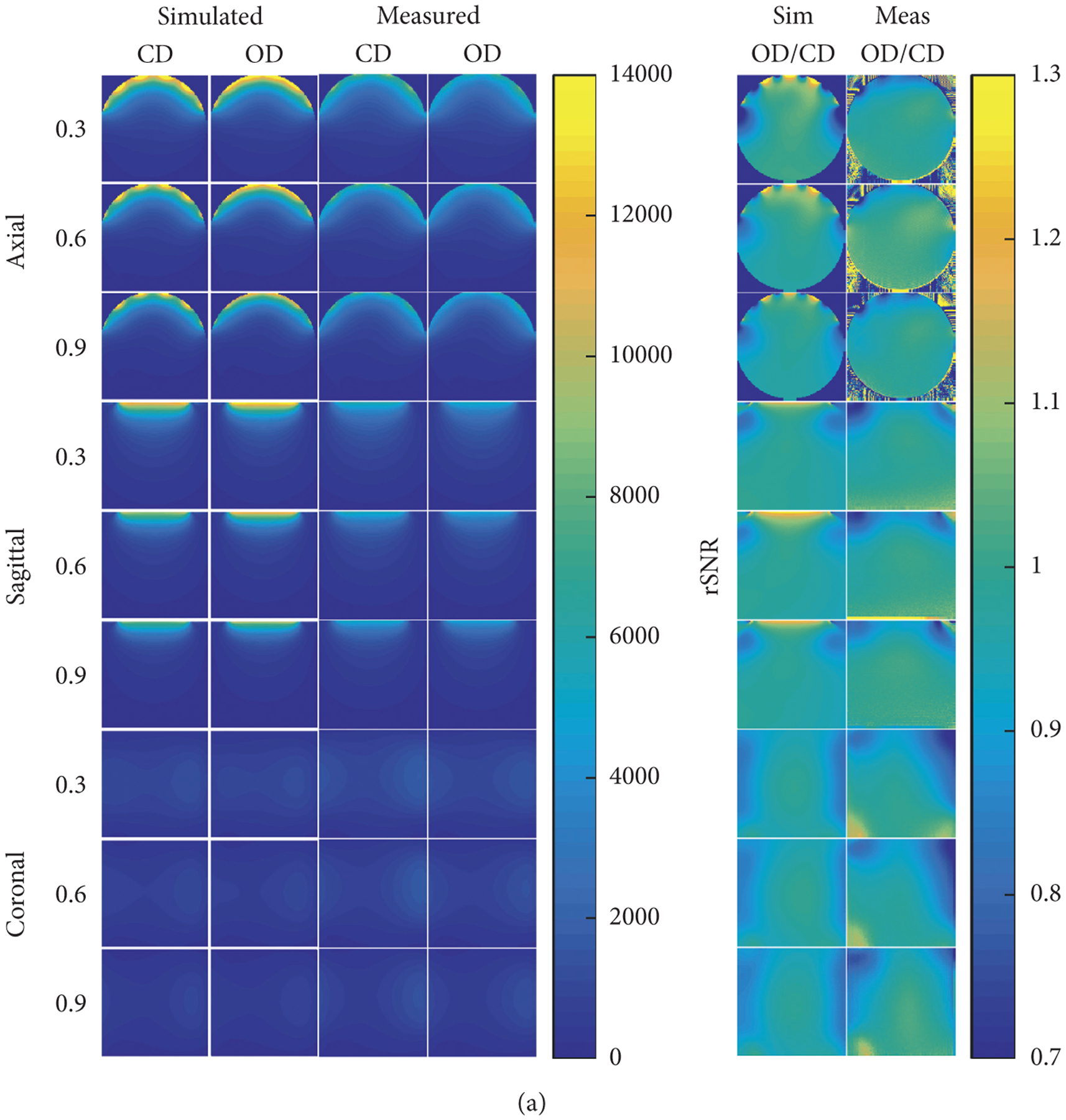

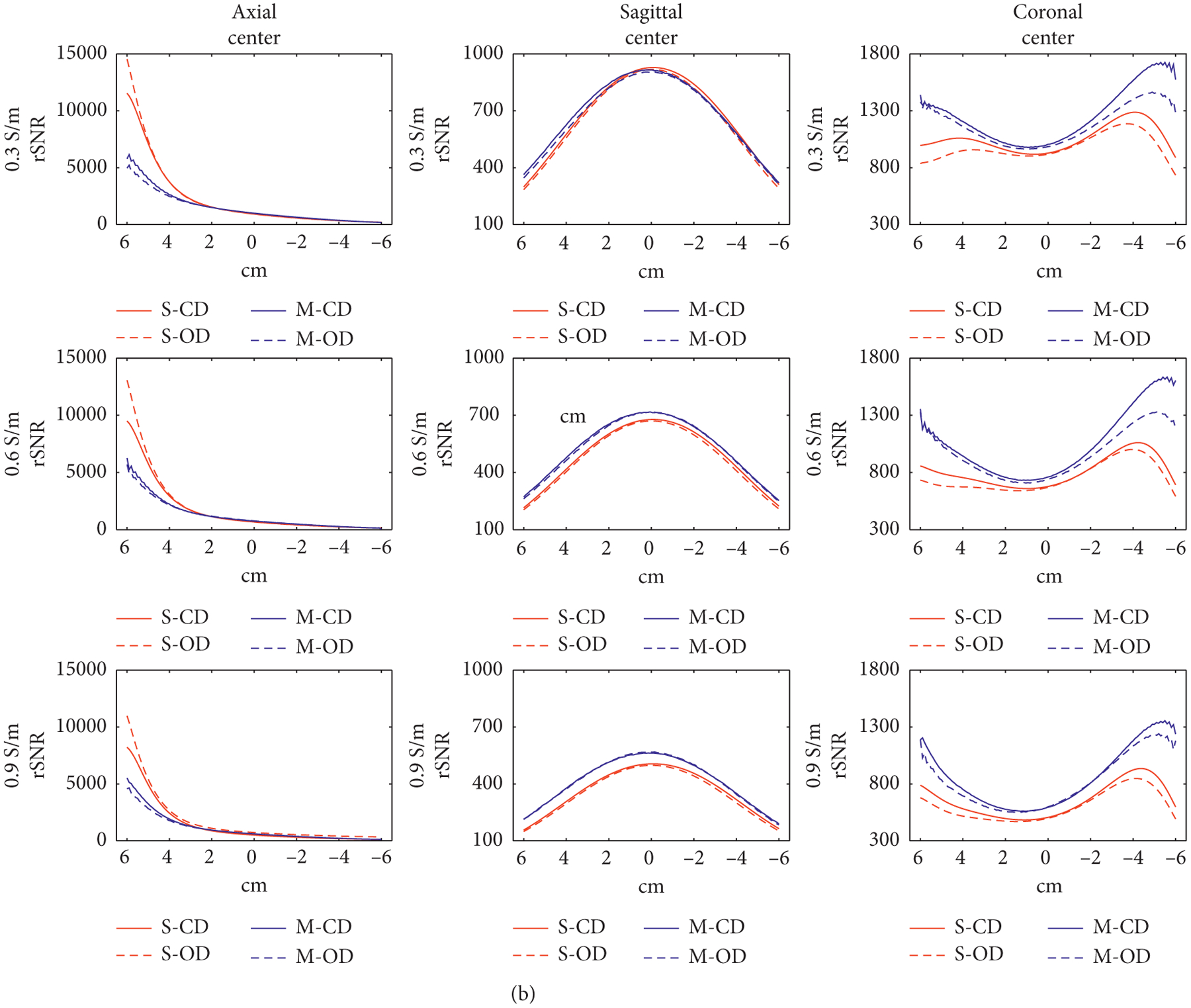
(a) Axial, sagittal, and coronal 2D SNR comparison between the 5-ch CD PA coil and 5-ch OD PA coil on 0.3, 0.6, and 0.9 S/m phantoms. All coil elements were tuned and matched to the 0.6 S/m phantom only. 2D images were acquired through the center of the phantom. (b) Axial, sagittal, and coronal 1D SNR comparison between the 5-ch CD PA coil and 5-ch OD PA coil on 0.3, 0.6, and 0.9 S/m phantoms. The legend is the same for all plots, and all coil elements were tuned and matched to the 0.6 S/m phantom only. 1D SNR plots were taken from the center of the 2D SNR images. The 1D SNR plots represent SNR data collected in the phantom along the *y*-axis, *z*-axis, and *x*-axis for the axial, sagittal, and coronal plots, respectively. See Figure 1 for axis reference.

Simulated and measured axial, sagittal, and coronal 2D SNR images, Figure 3(a), and 1D SNR plots, Figure 3(b), for the 5-ch PA coils are shown in Figure 3. There was reasonable agreement between the SNR images calculated from the simulations and those obtained from experiments on the scanner. When viewing the SNR ratio images, disagreement could be seen in the superficial region beneath the coil elements.

### 4.2. 5-ch PA Coils: Noise Correlation.

**Figure 4:**
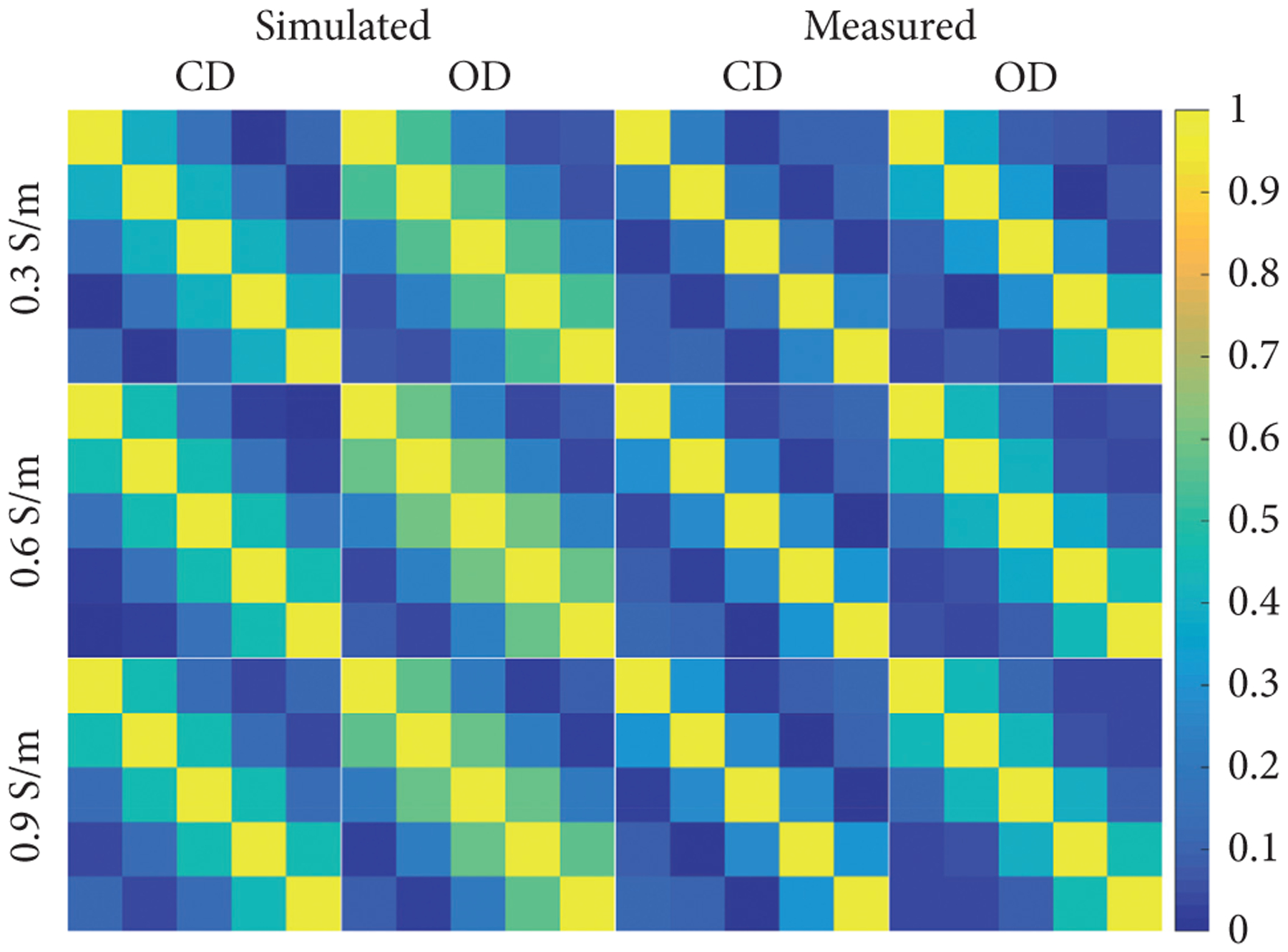
Correlation matrices simulated and measured on 0.3, 0.6, and 0.9 S/m phantoms using the 5-ch CD PA coil and 5-ch OD PA coil.

The corresponding noise correlation plots are shown in Figure 4. The maximum magnitude of the off diagonal values for the simulated 5-ch CD PA coil, rounded to the second decimal place, was 0.38, 0.41, and 0.40 and the simulated 5-ch OD PA coil values were 0.53, 0.55, and 0.54 for the 0.3, 0.6, and 0.9 S/m phantoms, respectively. The measured values for the 5-ch CD PA coil were 0.25, 0.30, and 0.32 and for the 5-ch OD PA coil were 0.40, 0.45, and 0.46 for the 0.3, 0.6, and 0.9 S/m phantoms, respectively.

### 4.3. 5-ch PA Coils: Parallel Imaging.

**Figure 5:**
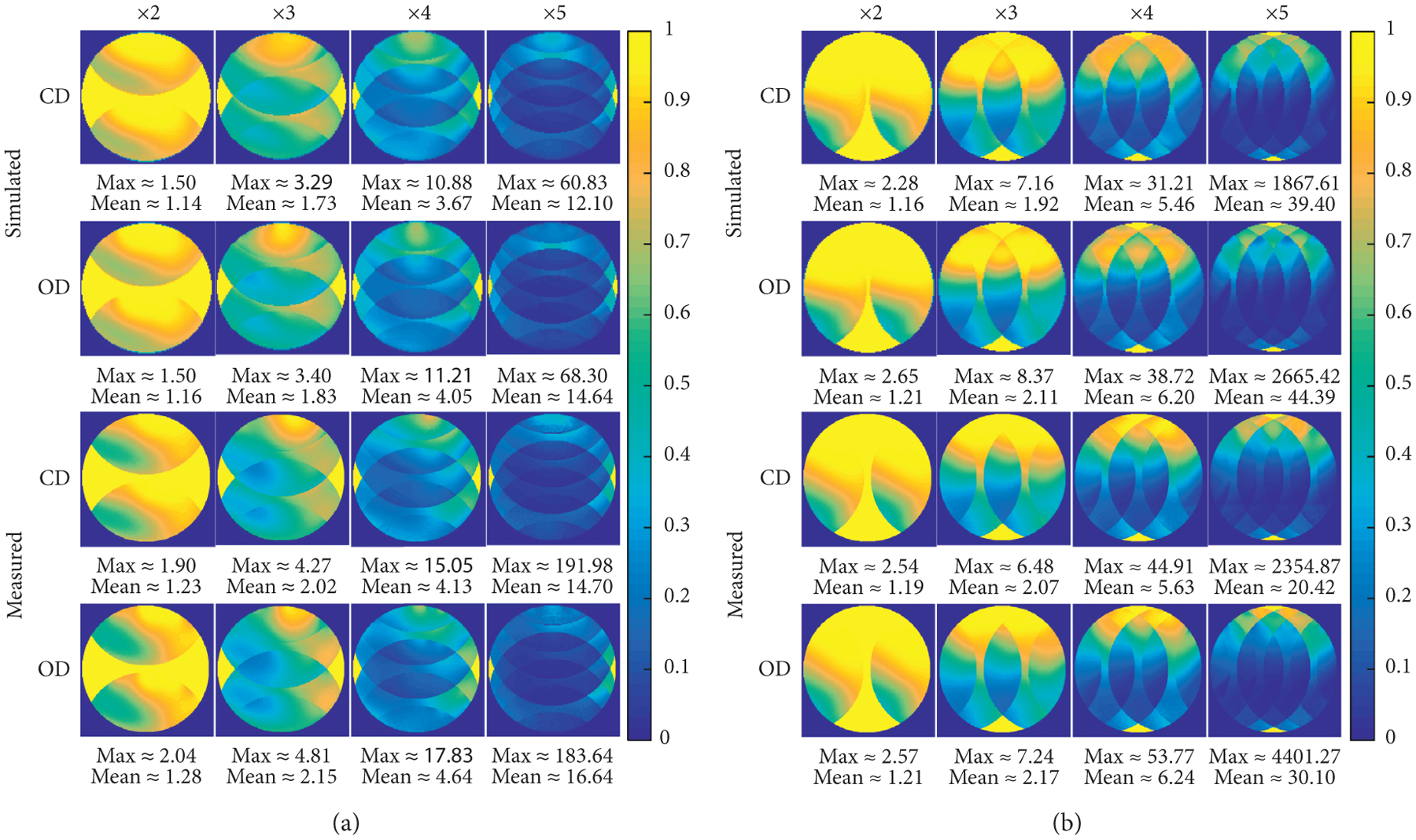

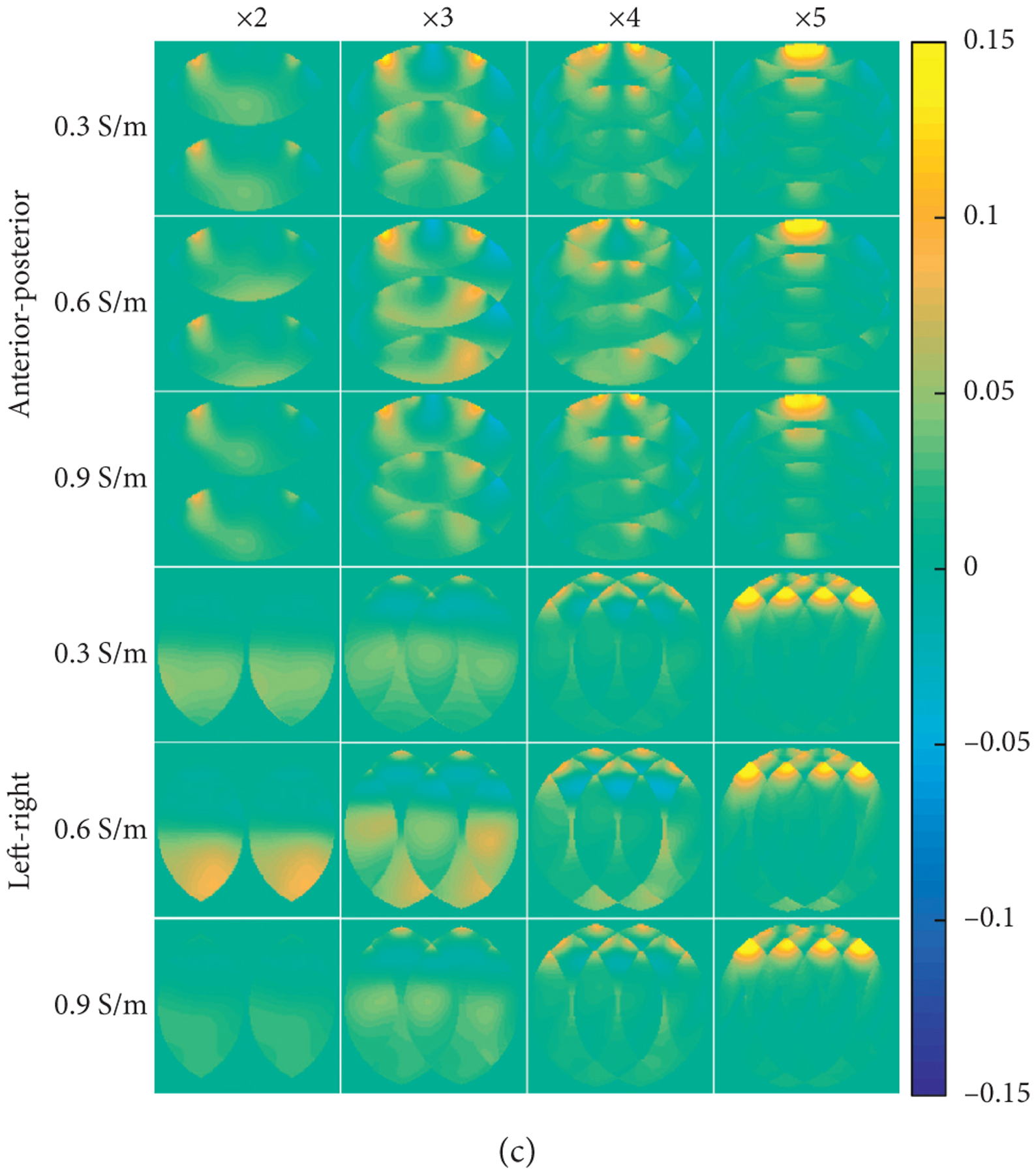
(a) 1/g-factor maps (anterior-posterior) for the 5-ch CD PA coil and 5-ch OD PA coil on the 0.6 S/m phantom. The maximum and mean g-factor values are listed below each figure. (b) 1/g-factor maps (left-right) for the 5-ch CD PA coil and 5-ch OD PA coil on the 0.6 S/m phantom. The maximum and mean g-factor values are listed below each figure. (c) Difference between the 1/g-factor maps when subtracting the 5-ch OD PA coil from the 5-ch CD PA coil 1/g-factor maps on the 0.3, 0.6, and 0.9 S/m phantoms. This was done for the anterior-posterior and left-right directions.

Parallel imaging results are shown in Figure 5. The 1/g-factor maps in the anterior-posterior and left-right directions are shown in Figures 5(a) and 5(b). Simulated differences in the 1/g-factor maps, by subtracting the 5-ch OD PA coils from the 5-ch CD PA coils 1/g-factor maps, are shown in Figure 5(c). The max and mean g-factor values are shown in Table 1. The 5-ch CD PA coil has better overall parallel imaging performance.

### 4.4. 5-ch PA Coils: S-Parameters.

The measured S-parameters of the 5-ch PA coils are shown in Table 2. These measurements were made at the preamplifier input. All preamps were installed except for those required to be removed for the measurement. Using a network analyzer, the S2, 1 value for each S-parameter was measured.

### 4.5. 5-ch PA Coils: Quality Factor.

The measured quality factors of the 5-ch PA coils are shown in Table 3. These values were measured on the completed coils with and without the phantoms. The preamp for the measured coil element was removed, and a probe, lightly coupled to that coil element, was used to make S2, 1 measurements. These S2, 1 measurements allowed the extraction of the quality factor.

### 4.6. 2-ch PA Coils: Coil Element Capacitor Value Variation.

Capacitance value results are shown in Table 4. The location of each capacitor with respect to each coil element is shown in Figure 2. The coil element variation in capacitor values was compared by taking the ratio of the match and tune capacitors. The quotients for the maximum and minimum capacitor value ratios are 3.28 (tune *C*_d_), 1.68 (tune *C*_t_), and 1.38 for the large 2-ch CD PA coil and large 2-ch OD PA coil tune configurations, respectively, and 3.28 (tune *C*_d_), 2.44 (tune *C*_t_), and 2.22 for the small 2-ch CD PA coil and 2-ch OD PA coil tune configurations, respectively.

## 5. Discussion

This paper used simulations and physical measurements to compare the relative performance of two methods of decoupling adjacent coil elements in receive RF PA coils as sample load varies. For both simulation and experiment, the PAs of each decoupling method were constrained to have the same number of coil elements and to have the same outer dimensions. Under those constraints with five total elements formed on a tissue mimicking cylindrical phantom, both the simulation and the measurement data show that there is very little difference in SNR between the two different PAs that were constructed with the two different decoupling methods.

The change in SNR ratio between the 5-ch CD PA coil and 5-ch OD PA coil, as sample load varies, is largely due to each array having different sized coil elements. When comparing a SNR ratio image calculated from either the 0.3 or 0.9 S/m phantom to that calculated from the 0.6 S/m phantom, as can be seen from Figure 3, the relative performance of the 5-ch OD PA coil increases as conductivity is decreased. This is observed despite the fact that the arrays were tuned and matched at 0.6 S/m. This observation most likely occurs because PA coils must be sample noise dominant to reach the ultimate intrinsic SNR [30]; otherwise, the coil resistance will become an additional source of uncorrelated noise [22]. The coil element resistance of the two 5-ch PA coils stays constant, whereas the total resistance increases ~230% from the 0.3 to the 0.9 S/m phantom for both 5-ch PA coils. This increase is almost entirely due to increases in sample resistance. Though the percentage increase in total resistance is nearly the same for the two 5-ch PA coils, the ratio of the sample to coil resistance is not. As conductivity decreases, the sample resistance of the 5-ch OD PA coil decreases at a faster rate than that of the 5-ch CD PA coil, because of the larger loop size, but sample noise dominance is still maintained. The larger drop in sample noise of the 5-ch OD PA coil results in the relative performance increase as conductivity decreases.

The CD method can be utilized in PA coil designs with more complex layouts [31–35]. A complex layout requires that at least one tune capacitor be available to tune each coil element. As an example, the inner three coil elements, of the 5-ch CD PA coil, each have one less tune capacitor than the outer coil elements, yet they still have at least one tune capacitor. In some cases, additional decoupling methods may need to be utilized; as seen in the work by Elabyad et al., where two equal capacitors and a gap were used to decouple coil elements that would otherwise not be left with a tuning capacitor if solely decoupled with the CD method.

The coil element capacitor value variation results help illustrate problems that could result from the reduced number of tune capacitors when using the CD method. One being that the CD method would most likely encounter unbuildable 2-ch PA coil configurations due to unrealizable capacitance values. Examples that would result in unrealizable capacitance values are match capacitance values that are lower than any capacitance value sold commercially or infinitely large capacitances required for proper tune and matching of coil elements because the match capacitance value is lower than the value of the equivalent tune capacitance. An approximation of the equivalent tune capacitance, required to tune a coil element, can be determined by calculating the equivalent capacitance of all the capacitors around that coil element, including the match capacitor. The match capacitor value decreases as sample resistance increases and both decoupling methods can have unrealizable match capacitances if the coil elements in the 2-ch PA coils are large enough or the conductivity of the sample is high enough for this to occur. The larger size of the 2-ch OD PA coil elements makes them more susceptible to this happening. Outside of large sample resistances, there are vast amounts of configurations that could potentially result in unrealizable PA configurations, but 2-ch CD PA coils in tune Cd configurations would be the most likely to encounter this problem because of their large quotient values. The larger the quotient values, the more likely that a single capacitance around the coil element will have a value lower than or close to the equivalent tune capacitance.

The overall parallel imaging performance is better for the 5-ch CD PA coil. This is to be expected since coil elements are not overlapped when using the CD method. Even in the cases where the 5-ch CD PA coil max g-factor values are larger than those of the 5-ch OD PA coil, the mean g-factor value of the 5-ch CD PA coil is still less than that of the 5-ch OD PA coil. As the acceleration rate increased, the difference in performance of the 5-ch CD PA coil increased over that of the 5-ch OD PA coil. This was especially true in the left-right direction. The g-factor values are better in the anterior-posterior direction when compared to those of the left-right direction. It would be expected that the left-right direction would have better imaging performance than that of the anterior-posterior direction, but in our case, the anterior-posterior direction is better due to the curvature of the PA coils.

The noise correlation values, of adjacent coil elements, are less for the 5-ch CD PA coil compared to those of the 5-ch OD PA coil for all phantoms. Correlation between coil elements is mainly determined by their geometry, which is why the change in correlation is so slight between the phantoms. The slight changes are the result of electric field differences between the phantoms. Also, the noise correlation values calculated in these simulations would be smaller if uncorrelated noise from the preamplifier and receive chain of the MRI scanner had been included.

In general, the process of building a CD or OD PA coil is quite similar. All of the copper traces/wire can typically be laid out for both PAs before tuning and matching begins. Once the capacitors are populated, the overlap distance and decoupling capacitance value between two adjacent coil elements are determined by minimizing S21 when both coil elements are tuned and matched to the same impedance. Wire bending or slight trace position adjustment can be used to minimize S21 for the overlap decoupling method, with the initial overlap distance preferably being close to the final distance. Using an adjustable capacitor, the capacitance value for the CD method can be found the same way as the overlap distance, except for the capacitor value is adjusted instead of the overlap distance. Once S21 is minimized for the two PA coils, it is an iterative process to finish the tune and matching of all PA coil elements.

The theory discussed previously in the theory section can be applied to the CD and OD methods at higher field strengths [36–39]. The theory is valid for higher field strengths with the consideration that electric coupling, assumed negligible in our case, may need to be considered in addition to magnetic coupling [16].

There is more work to be done before making generalized conclusions about the two decoupling methods. This study is limited by only having PA coils with five rectangular-shaped coil elements that are only tuned to the 0.6 S/m phantom before being tested on the 0.3 and 0.9 S/m phantoms. The only phantoms used are cylindrical phantoms with 12 cm diameters. There are many other test cases to be explored, so future work could include PA coils with channel counts other than five, coil elements of different shapes and sizes, phantoms with varying shapes and sizes, and testing the PA coils performance when the PA coils are initially tuned and matched to a phantom with a conductivity other than 0.6 S/m.

## 6. Conclusion

The results of this paper demonstrate that as the sample load varies, the 5-ch CD and OD PA coils are very similar in relative SNR and this similarity continues for the 1/g-factor of parallel imaging. The 5-ch CD PA coil appeared, in fact, to achieve slightly higher SNR and parallel imaging performance than the 5-ch OD PA coil in many areas. When deciding between a CD or OD PA coil, the designer needs to be aware that there are some configurations of the CD PA coil that will not be realizable due to fewer tune capacitors when compared to an OD PA coil of similar dimensions.

## Figures and Tables

**Table 1:** Max (mean) g-factor values (see Figures 5(a) and 5(b)).

		Anterior-posterior				Left-right			
	0.3 S/m	×2	×3	×4	×5	×2	×3	×4	×5
Simulated	CD	1.47	2.96	11.68	65.30	4.43	9.59	37.80	7142.59
		(1.15)	(1.78)	(3.92)	(13.74)	(1.30)	(2.37)	(6.76)	(43.88)
	OD	1.55	3.09	11.72	78.04	5.15	11.61	43.39	2775.42
		(1.17)	(1.87)	(4.30)	(16.51)	(1.36)	(2.58)	(7.39)	(45.69)
Measured	CD	1.66	3.55	15.14	847.96	5.82	10.80	79.26	2256.00
		(1.23)	(2.00)	(4.21)	(15.25)	(1.30)	(2.49)	(6.14)	(18.01)
	OD	1.79	4.09	17.04	506.96	6.34	12.79	82.29	4480.72
		(1.28)	(2.14)	(4.80)	(18.21)	(1.32)	(2.64)	(6.62)	(22.08)
	0.6 S/m	×2	×3	×4	×5	×2	×3	×4	×5
Simulated	CD	1.50	3.29	10.88	60.83	2.28	7.16	31.21	1867.61
		(1.14)	(1.73)	(3.67)	(12.10)	(1.16)	(1.92)	(5.46)	(39.40)
	OD	1.50	3.40	11.21	68.30	2.65	8.37	38.72	2665.41
		(1.16)	(1.83)	(4.05)	(14.64)	(1.21)	(2.11)	(6.20)	(44.39)
Measured	CD	1.90	4.27	15.05	191.98	2.54	6.48	44.91	2354.87
		(1.23)	(2.02)	(4.13)	(14.70)	(1.19)	(2.07)	(5.63)	(20.42)
	OD	2.04	4.81	17.83	183.64	2.57	7.24	53.77	4401.27
		(1.28)	(2.15)	(4.64)	(16.64)	(1.21)	(2.17)	(6.24)	(30.10)
	0.9 S/m	×2	×3	×4	×5	×2	×3	×4	×5
Simulated	CD	1.66	3.94	11.97	59.15	1.73	6.28	29.63	992.37
		(1.14)	(1.77)	(3.73)	(11.51)	(1.11)	(1.76)	(4.98)	(31.86)
	OD	1.64	3.93	12.22	61.17	1.80	6.98	34.27	925.98
		(1.16)	(1.84)	(3.98)	(13.32)	(1.13)	(1.86)	(5.40)	(32.28)
Measured	CD	2.14	4.86	13.54	167.99	1.89	5.71	67.80	3620.02
		(1.23)	(2.00)	(4.06)	(13.04)	(1.12)	(1.90)	(5.37)	(24.52)
	OD	2.25	5.25	16.65	177.03	1.93	6.29	71.22	4881.34
		(1.29)	(2.13)	(4.48)	(14.87)	(1.14)	(1.99)	(5.80)	(30.53)

**Table 2:** Simulated (S) versus measured (M) S-parameters of the 5-ch CD PA coil and the 5-ch OD PA coil.

El#	2	3	4	5
S/m	0.3 (0.6) 0.9	0.3 (0.6) 0.9	0.3 (0.6) 0.9	0.3 (0.6) 0.9
1 S-CD	−14.3 (−13.4) −13.4	−5.30 (−7.90) −9.70	−12.1 (−16.0) −18.8	−12.9 (−17.6) −21.5
S-OD	−12.1 (−10.5) −10.3	−4.10 (−6.00) −7.50	−10.1 (−13.3) −15.6	−10.8 (−15.1) −18.6
M-CD	−16.8 (−15.3) −14.4	−6.80 (−9.30) −10.7	−13.3 (−17.6) −20.0	−15.0 (−19.4) −21.9
M-OD	−11.2 (−11.4) −9.70	−5.70 (−7.80) −9.00	−12.7 (−17.5) −20.1	−9.10 (−13.5) −15.8
2 S-CD		−14.5 (−13.6) −13.5	−5.50 (−8.10) −10.0	−12.1 (−16.0) −18.8
S-OD		−12.5 (−10.6) −10.2	−4.00 (−5.90) −7.30	−10.0 (−13.3) −15.6
M-CD		−20.4 (−17.0) −16.6	−7.70 (−10.4) −12.0	−15.0 (−18.1) −20.6
M-OD		−14.7 (−12.3) −11.9	−5.80 (−8.60) −10.2	−12.4 (−16.6) −19.1
3 S-CD			−14.5 (−13.6) −13.5	−5.40 (−7.90) −9.70
S-OD			−12.6 (−10.6) −10.2	−4.00 (−6.00) −7.50
M-CD			−17.0 (−16.0) 15.7	−7.80 (−9.70) −10.9
M-OD			−15.2 (−13.0) −12.1	−5.60 (−7.70) −8.60
4 S-CD				−14.2 (−13.4) −13.4
S-OD				−12.2 (−10.5) −10.3
M-CD				−16.7 (−15.4) −14.0
M-OD				−11.0 (−11.8) −9.60

**Table 3:** Quality factor of the 5-ch CD PA coil and the 5-ch OD PA coil.

	5-ch CD PA coil					5-ch OD PA coil				
	*Q*_U_		*Q*_L_		*Q*_U_/Q_L_	*Q*_U_		*Q*_L_		*Q*_U_/Q_L_
S/m El#	0.0	0.3	0.6	0.9	0.3 (0.6) 0.9	0.0	0.3	0.6	0.9	0.3 (0.6) 0.9
1	192	88	61	49	2.2 (3.1) 3.9	160	74	55	45	2.2 (2.9) 3.6
2	173	81	54	43	2.1 (3.2) 4.0	140	66	48	40	2.1 (2.9) 3.5
3	173	80	60	51	2.2 (2.9) 3.4	140	76	56	49	1.8 (2.5) 2.9
4	183	78	55	47	2.4 (3.3) 3.9	165	74	50	40	2.2 (3.3) 4.1
5	185	85	59	49	2.2 (3.1) 3.8	160	84	60	46	1.9 (2.7) 3.5

*Note*. Measurements made on completed coils.

**Table 4:** Capacitor value comparison (see Figure 2).

Small (pF)	*C*_max_/*C*_min_	*C*_m1_	*C*_m2_	*C*_t1_	*C*_t2_	*C*_d_	*C*_t_/*C*_d_	*C*_equivalent_
Capacitive								
(tune *C*_d_)	3.28	137.7	137.7	42.03	42.03	84.75	84.75	~18.30
(tune *C*_t_)	2.44	137.5	137.5	56.26	56.26	84.75	56.26	~18.31
Overlap	2.22	144.8	145.3	65.24	65.06	—	—	~18.91
Large (pF)								
(tune *C*_d_)	3.28	69.45	69.45	21.15	21.15	75.00	75.00	~8.69
(tune *C*_t_)	1.68	68.65	68.65	40.97	40.97	75.00	40.97	~8.74
Overlap	1.38	69.70	68.60	50.60	50.59	—	—	~8.84

## Data Availability

The data used to support the findings of this study are available on request from either J. Rock Hadley at rock.hadley@utah.edu or Michael J. Beck at mjb27@utah.edu.
